# First Isolation and Molecular Characterization of Pseudorabies Virus in a Hunting Dog in Sicily (Southern Italy)

**DOI:** 10.3390/vetsci8120296

**Published:** 2021-11-29

**Authors:** Jessica Maria Abbate, Alessia Giannetto, Carmelo Iaria, Kristian Riolo, Giuseppe Marruchella, Jasmine Hattab, Placido Calabrò, Giovanni Lanteri

**Affiliations:** 1 Department of Veterinary Sciences, University of Messina, Polo Universitario Annunziata, 98168 Messina, Italy; jabbate@unime.it; 2 Department of Chemical, Biological, Pharmaceutical and Environmental Sciences, University of Messina, Polo Universitario Papardo, 98166 Messina, Italy; agiannetto@unime.it (A.G.); kririolo@unime.it (K.R.); glanteri@unime.it (G.L.); 3 Faculty of Veterinary Medicine, University of Teramo, Loc. Piano D’Accio, 64100 Teramo, Italy; gmarruchella@unite.it (G.M.); jhattab@unite.it (J.H.); 4 Veterinary Practitioner, 98127 Messina, Italy; placido.calabro@libero.it

**Keywords:** pseudorabies, PrV, dog, SHV-1, Aujeszky’s disease, Sicily

## Abstract

Pseudorabies virus (PrV) is the etiological agent of Aujeszky’s disease, a viral infection that causes neurological lethal illness in mammals other than swine. Herein, we describe the occurrence of PrV infection in a hunting dog that had been bitten by an infected wild boar in Sicily, reporting for the first time genetic and phylogenetic data on the virus strain isolated in a dog in this Italian region. The dog was referred for severe neurological signs, respiratory distress, and intense itch around the muzzle. Death occurred within 48 h to the onset of clinical signs. On gross examination, self-induced skin lesions to the head due to intense itching and diffuse cerebral congestion were observed, whereas mild, aspecific, nonsuppurative meningitis was histologically diagnosed. Diffuse PrV positivity in neurons of the brainstem was observed by immunohistochemistry. PrV DNA was isolated and amplified from olfactory bulbs by nested PCR, targeting the viral glycoprotein G gene, and the sequence obtained matched with sequences of PrV isolates from dogs and wild boar. Isolation of PrV in the dog herein analysed denotes the spread of the virus in wild boar populations in Sicily and provides a proof of direct interspecies transmission. Thus, there is an urgent need to increase our understanding of the epidemiology of the PrV infection in wildlife to provide tools to trace possible spill over into domestic pigs or other livestock.

## 1. Introduction

Pseudorabies (Aujeszky’s disease) is an OIE-listed, notifiable, highly contagious disease that causes great economic losses to the swine industry worldwide. 

Suid herpesvirus 1 (SuHV-1), also called pseudorabies virus (PrV) or Aujeszky’s disease virus (ADV), is the causative agent of the disease: an enveloped, double-stranded, linear DNA virus, which belongs to the family Herpesviridae, subfamily Alphaherpesvirinae [1]. Pseudorabies virus seems to be a potential zoonotic pathogen and responsible for encephalitis and endophthalmitis in humans, thought to be refractory to the infection for a long time [2,3]. 

Members of the Suidae family are the natural hosts for SuHV-1 and the only species that could survive a productive infection, becoming reservoir hosts and source of infection for susceptible mammals, including humans [1,2,3,4]. In natural hosts, the outcome of the disease varies according to animals’ age, level of passive and active immunity, the virulence of the strains involved, and the dose of virus responsible for infection [5]. Old animals generally show only mild respiratory clinical signs and lower body weight gain although clinically inapparent infection commonly occur. Conversely, PrV causes reproductive syndrome in pregnant sows and high mortality rate in young piglets due to severe encephalomyelitis [5,6]. Furthermore, PrV is a highly neurotropic virus, reaching the nervous system by trans-synaptic passage and retrograde axonal transportation and leading to latent infection in pigs, which survive acute infection [5].

Extensive vaccination programs in swine herds have successfully led to the eradication of the disease in most of the European countries. However, PrV still remains widespread in populations of non-domestic swine, and the circulation and the persistence of PrV in several ecological settings pose severe constrains in disease eradication programs [4,7,8,9,10]. Especially, wild boars (*Sus scrofa* L. 1758) and feral swine serve as persistent reservoirs for PrV in Europe [11,12,13], and interestingly, the detection of seropositive wild boars seems to indicate that PrV has found an ecological niche already in times when eradication programs in swine herds were established in European countries. Of note, for the two past decades, there has been a prominent increase in PrV seroprevalence in wild reservoirs in most of the European countries also in accordance with continuously expanding wild boar populations [4]. In Italy, although infection prevalence rates have been significantly reduced since the beginning of the PrV-monitoring program in 1997 (DM 1 April 1997) [1,7], Aujeszky’s disease has not been completely eradicated from swine herds to date. Additionally, PrV remains widespread in sylvatic reservoirs hosts, as denoted by numerous surveillance studies, with the highest seroprevalence value recorded in Central Italy [7,8,9,14]. 

Although wild-boar-adapted PrV strains are potentially less pathogenic also in domestic pigs [15], they are highly pathogenic strains for several susceptible domestic and sylvatic mammals that share the same territories [16]. Indeed, wild-boar-adapted PrV strains have been isolated in cattle, in wild carnivores (e.g., fox, wolf), as well as in dogs used for wild boar hunting [8,17,18,19,20]. Generally, PrV infection is acquired through direct contact with natural hosts/reservoirs by fecal-oral or aerosol routes, through bites [21], or consuming contaminated carcasses of infected pigs or wild boars. Interestingly, also the exposure to the live vaccines employed for domestic swine may induce the disease in foxes [22] as well as in sheep [23] and dogs [24]. In non-natural hosts, the infection is usually fatal, especially in canids, in which death occurs within 24–48 h, preceded by severe neurological clinic signs and a characteristic pruritus of the head, known as “mad itch” [25]. 

In this paper, we report the occurrence of Pseudorabies in a hunting dog that had been bitten from a wild boar in the Province of Messina (Sicily region; Southern Italy). Pseudorabies was suspected based on anamnestic data and clinical course and further confirmed by immunohistochemistry, PCR, and sequence analysis. To the best of the authors’ knowledge, this is the first report in which PrV infection in dog has been molecularly investigated in this region so far.

## 2. Materials and Methods

### 2.1. Cases Presentation 

In February 2021, two 2-year old, female crossbreed dogs were referred to a private veterinary hospital in Messina (Sicily; Southern Italy) for severe neurological signs, respiratory distress, and intense itch around the muzzle and ears. The owner indicated those dogs had been used for hunting a few days before the onset of clinical signs, receiving multiple bites from a wild boar. At clinical examination, animals showed similar symptoms, which included trismus, tetraparesis, generalized muscular spasms, sialorrhea, dyspnea, and self-inflicted lesions of the skin, probably due to intense itch. Dogs underwent conservative treatment, and death occurred within 24–48 h to the onset of clinical signs. Based on anamnestic data and clinical course, Pseudorabies was suspected, and the owner decided to send only one dog to the Department of Veterinary Science of the University of Messina to achieve a definitive diagnosis. 

### 2.2. Histopathology 

During necropsy, representative portions of all organs were sampled, fixed in 10% neutral buffered formalin, and embedded in paraffin for histopathological investigations; in addition, representative portions of central nervous system (brain, brainstem, cerebellum) were sampled and stored at –80 °C for genomic DNA extraction. 

Paraffin-embedded, 5-μm tissue sections were stained with hematoxylin and eosin (HE) for histopathological examination and visualized using a Leica DM6B microscope (Leica Camera, Wetzlar, Germany) using Leica Application Suite X Software(Leica Microsystems GmbH, Wetzlar, Germany), and images were acquired using a Leica DFC 7000 T(Leica Camera, Wetzlar, Germany). 

### 2.3. Immunohistochemistry 

Immunohistochemistry (IHC) was performed on paraffin-embedded tissue sections of selected organs (brain, brainstem, olfactory bulbs), using a rabbit polyclonal antibody anti-pseudorabies virus (Abcam 3534, Cambridge, UK). Primary antibody was incubated overnight at room temperature (18–20 °C) at the final dilution of 1:100. Tissue sections were previously heat-treated for antigen retrieval (microwave oven at 600 W, 3 × 5 min in citrate buffer 0.01 M pH 6.0), and immune reactions were revealed by means of an Avidin-biotin complex (ABC) detection method (Vector Laboratories Inc., Burlingame, California), and 3,3′-Diaminobenzidine (DAB) was used as chromogen. Slides were counterstained with Papanicolaou’s hematoxylin. Suitable positive and negative controls were included in IHC reactions. 

### 2.4. Viral Nucleic Acids Extraction 

Genomic DNA extraction from the nervous system tissues (olfactory bulbs and brain) was performed with the Nucleo Spin Plant II kit (Macherey-Nagel), according to manufacturer’s instructions. The contaminating RNA was removed via RNase (200 µg/mL) treatment. UV absorbance at 260, 280, and 230 nm was measured by NanoDrop 2000 (Thermo Scientific; Wilmingtom, MA, USA) to verify DNA quantity and purity. The DNA extracted samples were stored at –20 °C until needed. 

### 2.5. Polymerase Chain Reaction (PCR) and Sequencing

The presence of PrV was verified by nested PCR targeting the viral glycoprotein G (gG) gene using the following primer sets, whose reliability had been previously tested by Yoon et al. (2005) [26]: PRV_G_F1: ATGTTGTCGTTTGATCCCGTC; PRV_G_R1: AGCCGCGAGAGTAGTCCGTCC (product size 327 bp); PRV_G_F2: GAATGTGGACCGTATAAAACGGC; and PRV_G_R2: TGGCCGTAGCAGAGCTCC (product size 168 bp). The first PCR amplification was performed using 500 ng of genomic DNA and Taq DNA Polymerase Recombinant kit (Invitrogen) in a 25-µL reaction volume. The DNA was amplified in the Ep-Gradient Mastercycler (Eppendorf) with the following PCR conditions: after a first step of 95 °C for 10 min, DNA was subjected to 30 cycles of 94 °C for 45 s, 62 °C for 1 min, and 72 °C for 1 min, with a final extension of 72 °C for 10 min. A second nested PCR amplification was performed applying the same conditions and using 5 µL of the products of the first PCR.

A 2.0% agarose gel electrophoresis was performed to resolve the PCR products; then, the fragments of the expected size were purified using the E.Z.N.A Gel Extraction Kit (OMEGA), following the manufacturer’s protocol. DNA sequencing of the purified fragments was performed on the Applied Biosystems 3730 DNA Analyzer (Thermo Fisher Scientific), using the same primers used for amplification. 

### 2.6. Sequence Analysis 

The sequence results were analysed by BLASTN similarity search against the National Center for Biotechnology Information (NCBI; https://blast.ncbi.nlm.nih.gov/Blast.cgi; accessed on: 23 September 2021) database to calculate statistical significance of the matches found. Multiple sequence alignment of selected gG nucleotide sequences was performed using the ClustalW algorithm (https://www.genome.jp/tools-bin/clustalw; accessed on: 23 September 2021). The obtained gG sequence was aligned with related sequences from dog and wild boar (accession number AP018925.1 and KJ717942.1, respectively) previously deposited in GenBank. Following alignment of our PrV isolate with related sequences retrieved from Blast search and available in GenBank, phylogenetic analysis was carried out using the Neighbour Joining (NJ) method and the *p*-distance model using MEGA11 software [27] with bootstrap analysis involving 1000 replicates.

## 3. Results

### 3.1. Gross and Histopathological Findings 

**Figure 1:**
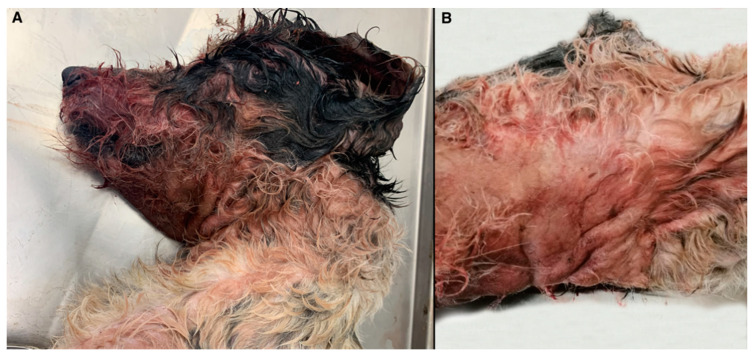
Gross examination of the infected dog. Self-inflicted cutaneous lesions around the muzzle, ears, and neck probably secondary to intense itching (**A**). Diffuse oedema was observed in the region of the neck (**B**).

On gross examination, self-inducted cutaneous lesions around the muzzle, ears, and neck were observed, including abrasions, ecchymoses, and hair loss (Figure 1A) probably due to intense scratching. Diffuse oedema was noted in the region of the neck (Figure 1B). Examination of organs in thoracic cavity revealed severe, diffuse, acute pulmonary congestion, and oedema, with multifocal hemorrhages and alveolar emphysema and acute, multifocal endocardial hemorrhages. Finally, severe and diffuse cerebral congestion was observed. 

**Figure 2:**
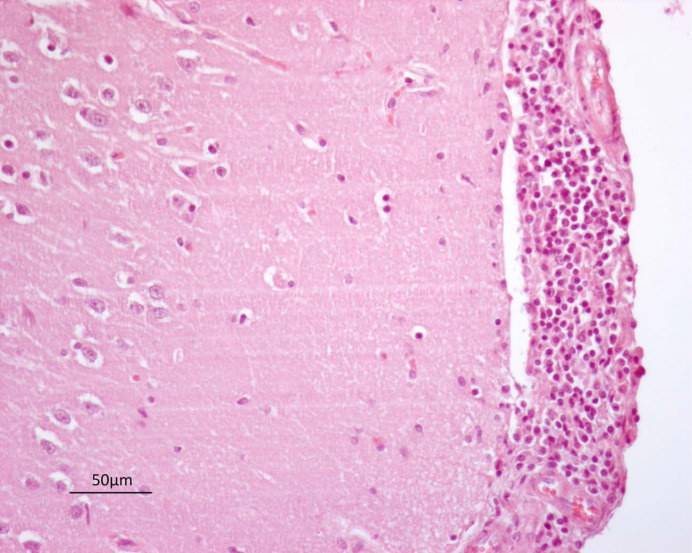
Brain. Mild, multifocal to coalescing infiltration of lymphocytes, plasma cells, and occasional histiocytes within the leptomeninges (20×).

On histology, a severe, diffuse lymphoplasmacytic dermatitis with extensive oedema was noted in the neck region. Histopathological examination of all examined areas of brain revealed a mild to moderate, multifocal to coalescing infiltration of lymphocytes; plasma cells; and occasional histiocytes within the leptomeninges, also expanding Virchow–Robin spaces as perivascular cuffs. In brain and brainstem, only a few neurons display hypereosinophilic shrunken cytoplasm, with pyknotic or karyorrhectic nuclei (necrosis), sometimes surrounded by proliferation of glial cells (satellitosis). Histopathological findings of the brain and brainstem were compatible with non-specific nonsuppurative meningitis (Figure 2). 

### 3.2. Immunohistochemistry

**Figure 3:**
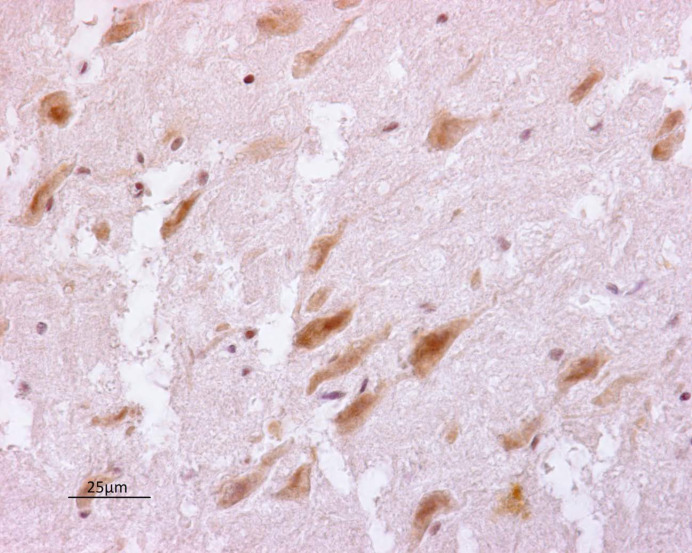
Brainstem. Pseudorabies virus positivity in neurons (arrow) (40×).

Polyclonal antibody anti-PrV reacted intensively in positive-control tissues, and equivalent staining was observed on positive tissue sections from the dog herein analyzed. A granular reddish to brownish staining was observed in the cytoplasm of neurons and dendritic and axonal processes (Figure 3). Nonspecific background staining did not interfere with the interpretation of specific staining. 

### 3.3. Molecular Identification of Pseudorabies Virus

The genomic DNA from the central nervous system of the hunting dog was successfully amplified by nested PCR only from olfactory bulbs, obtaining a fragment of 168 bp. The obtained nucleotide sequences were identical among samples, and one representative sequence was submitted to GenBank database under the accession number MZ667614 (isolate S21).

**Figure 4:**
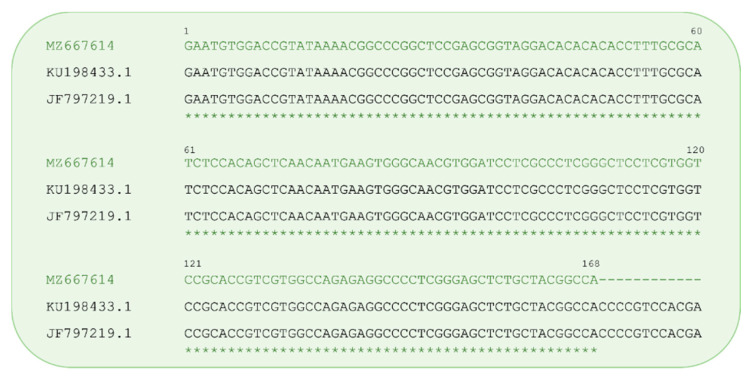
Alignment of the Pseudorabies virus gG sequence from this study (accession n. MZ667614) with related sequences obtained from *Canis lupus familiaris* (accession n. KU198433.1) and *Sus scrofa* (accession n. JF797219.1) previously deposited in GenBank. Asterisks indicate identity.

Blast search showed that our sequence matched with the several sequences of different isolates of SuHV-1 belong to genotype I with an E value of 1e-80, query cover, and percent identity of 100%. The identity of gG gene here identified with the SuHV-1 gG from *Canis lupus familiaris* (accession numbers: KU198433.1, KC981239.1, and AP018925.1 share the same nucleotide sequence in the analysed region) and from wild boar (accession number: JF797219.1) is showed in Figure 4.

**Figure 5:**
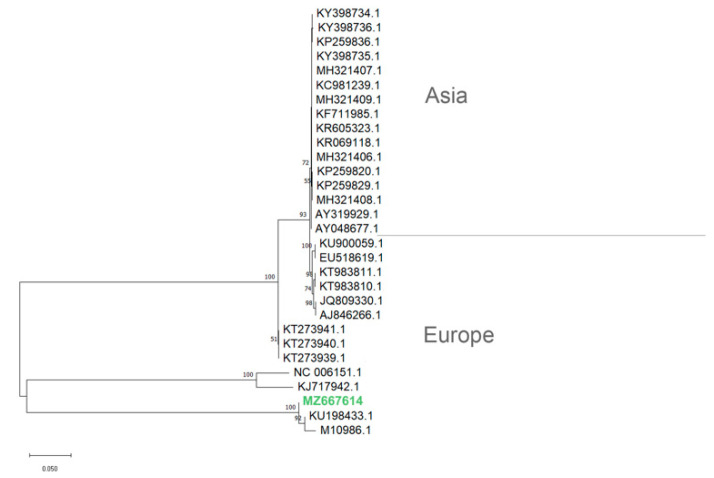
Phylogenetic tree (NJ) depicting relationships among the PrV isolates based on glycoprotein G genes. Nucleotide sequences were aligned using the ClustalW algorithm, and the evolutionary history was inferred using the Neighbor Joining (NJ) method in MEGA11 (Tamura et al., 2021). Bootstrap values (1000 replicates) are indicated at the nodes. GenBank accessions from PrV strains are as follow (accession/strain): KU198433.1/ADV32751/Italy2014; NC_006151.1/, KJ717942.1/ and AJ846266.1/Kaplan; KT273939.1/SuHV1/WB6/NIVS2014; KT273940.1/SuHV1/WB7/NIVS2014; KT273941.1/SuHV1/WB8/NIVS2014; JQ809330.1/DUL34Pass; KT983810.1/Hercules; KT983811.1/Kolchis; EU518619.1/ and KU900059.1/NiA3; MH321406.1/HN-HH, MH321407.1/HN-NY, MH321408.1/HN-NX, MH321409.1/HN-ZZ, KP259820.1/YY, KP259829.1/LY, KP259836.1/GA, KR069118.1/isolate BJ, KR605323.1/AH02LA, KF711985.1/Xiang A, KC981239.1/BJ/YT, KY398734.1/isolate SCT17-11, KY398735.1/isolate HuNF83-14, KY398736.1/isolate SDZD-16, M10986.1, AY319929.1/Ea, and AY048677.1.

The phylogenetic analysis showed that the herein identified gG gene of the PrV isolate from the hunting dog in Sicily clustered with the corresponding gG sequence of the PrV strain ADV32751/Italy2014 available in GenBank (KU198433.1) and previously isolated from a symptomatic dog in Italy (Figure 5). The phylogenetic tree of PrV isolates based on partial nucleotide gG sequences showed that PrV strains, whatever their host species, separates in distinct clades depending on the different geographical locations (i.e., Europe or Asia).

## 4. Discussion

In this study, we describe the occurrence of Pseudorabies in a hunting dog in Sicily (Southern Italy), where, to the best of the authors’ knowledge, no cases in dogs have been molecularly investigated. Pseudorabies was strongly suspected based on history of exposure to a wild boar, clinical course, and histopathological findings, and PrV infection was further confirmed by immunohistochemistry, viral DNA isolation, and sequence analysis. PrV was also considered to be the causative agent of disease, responsible for death in the other dog due to the same anamnestic data, symptoms, and clinical course although the carcass was not submitted for further investigation.

Pseudorabies is one of the most economically important disease of farmed pigs globally. Additionally, the disease represents a great concern for wild boar and feral pig populations, which act as persistent reservoirs for viral infection [11,12,13] and represent a potential threat for occasional animals, including dogs [4,10,16,28]. As matter of fact, cases of PrV infection in dogs are increasingly described worldwide [8,18,19,20], and PrV is generally acquired through direct contact with natural hosts, through bites [21], or consuming contaminated carcasses of reservoir hosts. Isolation of PrV DNA in dog herein investigated denotes the circulation of the virus in wild boars although epidemiological data are currently lacking. Additionally, isolation of PrV DNA provides a proof of direct interspecies transmission. Interestingly, the alignment of the gG sequences obtained in this study with the related sequences of PrV isolates from dogs and wild boar, available in GenBank, showed that they share the same nucleotide sequence, suggesting a potential PrV infection in dogs by contact with wild boar. Phylogenetic analyses based on the gG sequences showing that strains isolated from dogs can cluster with those related to wild boars and domestic pigs suggested that PrV transmission can occur from a different population. Moreover, although the small number of gG sequences deposited in GenBank, PrV stains separating in different clades strongly suggest that their geographical location, whatever their host species, is an important feature, as previously reported for gC proteins from PrV strains from Europe/America or Asia [29]. Of note, Sozzi et al. (2014) [1] offered an interesting insight into genomic characterization of Pseudorabies virus strains in Italy, demonstrating a clear differentiation between viral strains isolated from hunting dogs and then traced back to wild boars and those amplified from dogs in the farms, which were similar to strains affecting domestic pigs. Conversely, genomic characterization of PrV isolated from sylvatic reservoirs should help to identify the origin of infection, and this aspect will be worthy of future investigations. Moreover, phylogenetic analysis of available nucleotide sequences from Europe highlights the diversity among the PrV wild boar isolates as result of multiple introductions from domestic pigs into wildlife [10]. The genetic similarity among wild swine and domestic pig PrV isolates suggest that the transmission has taken place in both directions [10]. Interestingly, the area where hunting was carried out for the cases herein described (Nebrodi park; northeastern Sicily) is characterized by high density of wild boars, which live in contact with feral and domestic pigs.

As matter of fact, most of the domestic pigs in Sicily region are traditionally bred in extensive or semi-extensive farming system, sharing feeding and watering areas with wild boar populations and other livestock, with large opportunities of pathogens transmission [30,31]. In this area, the seroprevalence of SHV-1 in domestic pigs constantly increased in the last decade, reaching the highest value in 2017 (i.e., 7.8%) [31], raising the hypothesis that the transmission of PrV in wild boars took place from infected domestic pigs through continuous contact between the two species and contamination of grazing, feeding, and watering areas.

Di Marco Lo Presti V. et al. recently described a multi-species outbreak of Aujeszky’s disease occurred in Sicily in 1996, which involved wild and domestic animals, including two dogs [32]. The outbreak was probably related to the circulation of SHV-1 in wild boars and suggested the spread of the disease and the potential epizootic risk in multi-host context in this Italian region [32]. Therefore, much attention should be employed in controlling Aujeszky’s disease in natural reservoir hosts. Extensive vaccination programs, based on the use of gE-deleted live-attenuated vaccines, as well as epidemiological surveillance by intra-vitam serological diagnostic methods, quarantine, the elimination of infected animals and the application of severe biosecurity measures allow to efficiently control the spread of the disease in swine herds. Conversely, free-roaming zootechnical systems for domestic pigs in some areas of Sicilian region as well as uncontrolled circulation of the virus in wild boar populations make it difficult to apply adequate biosecurity measures and also poses several constrains to the national eradication programs for the disease.

For PrV cases herein described, the hunting dog showed clinical signs indicative for PrV infection, including the characteristic “mad itch”, a neuropathic pruritus at the point of virus inoculation and responsible for self-inflicted cutaneous lesions [33,34]. Additionally, Pseudorabies was suspected based on history of direct exposure to wild boar. The incubation time generally varies between 2–9 days, and the spread of PrV to the brain along nerve axons is responsible for the onset of neurological signs [20,25,33,35]. Dogs are dead-end hosts, lethally infected within 24–48 h of the onset of clinical signs, and no specific treatment for PrV infection is available [25]. Generally, Pseudorabies in dogs is not associated with specific gross lesions, excepted for mechanical trauma and automutilation due to the intense pruritus. In addition, histopathological findings are not specific for PrV infection [33]. PrV in dogs is responsible for non-specific nonsuppurative encephalitis, and the brainstem is frequently involved where glial nodules and occasional viral inclusion bodies are found in neurons and astrocytes [34]. Several etiological agents can cause meningitis and encephalitis in dogs, including viral (rabies virus; canine distemper virus), bacterial, mycotic and parasitic diseases, as well as various toxicosis [33]. Herein, rabies and canine distemper were excluded, as the hunting dogs had been regularly vaccinated, and histopathological findings (i.e., lymphoplasmacytic meningitis) allow us to rule out other causative agents (bacteria, parasites, mycosis). No toxicological testing was performed. However, based on non-specific gross and histopathological findings, the definitive diagnosis has been achieved, identifying viral antigens in the cytoplasm of infected cells by immunohistochemistry by molecular tools and sequence analysis.

## 5. Conclusions

This study describes for the first time the occurrence and molecular identification of PrV infection in a hunting dog, transmitted by an infected wild boar in the Sicily region. Considering the spread of PrV infections in wild boar populations, there is an urgent need to increase our understanding of the epidemiology of PrV infection in wildlife and to provide tools to trace possible spillover into domestic pigs or other livestock. Additionally, considering the recent reports on human susceptibility to PrV, the disease might be considered a full-fledged zoonosis, and much attention should be paid to the control of wild boar population.

## Data Availability

The data presented in this study are available in (Abbate, J.M.; Giannetto, A.; Riolo, K.; Iaria, C.; Marruchella, G.; Calabrò, P.; Lanteri, G., 2021). First isolation and molecular characterization of Pseudorabies virus in two hunting dogs in Sicily (Southern Italy). Vet. Sci. 2021, 8, 296. https://doi.org/10.3390/vetsci8120296, and sequence has been deposited in GenBank (Accession n. MZ667614; accessed on: 23 September 2021).
